# Discovery of a heat-generated compound DHD derived from *Patrinia villosa* water extract with inhibitory effects on colon cancer cells viability and migration

**DOI:** 10.3389/fchem.2023.1195883

**Published:** 2023-06-02

**Authors:** Huihai Yang, Tao Zheng, Chuen-Fai Ku, Cheuk Kit Ngai, Grace Gar-Lee Yue, Hung Kay Lee, Clara Bik-San Lau

**Affiliations:** ^1^ Institute of Chinese Medicine, The Chinese University of Hong Kong, Shatin, Hong Kong SAR, China; ^2^ State Key Laboratory of Research on Bioactivities and Clinical Applications of Medicinal Plants, The Chinese University of Hong Kong, Shatin, Hong Kong SAR, China; ^3^ Department of Chemistry, The Chinese University of Hong Kong, Shatin, Hong Kong SAR, China; ^4^ Li Dak Sum Yip Yio Chin R&D Centre for Chinese Medicine, The Chinese University of Hong Kong, Shatin, Hong Kong SAR, China

**Keywords:** *Patrinia villosa*, valerosidate, colon cancer, thermal hydrolysis, 8,9-didehydro-7-hydroxydolichodial

## Abstract

**Introduction:** The plant *Patrinia villosa* Juss. (PV) has long been used as a medicinal herb for treating intestinal disorders. Pharmacological activities such as anti-oxidation, anti-inflammation, and anti-cancer effects of compounds isolated from PV have been reported, but these bioactive compounds were not derived from PV water extract (PVW). Therefore, in the present study, we aimed to identify the active component(s) of PVW which exhibit inhibitory activities in colon cancer cells viability and migration.

**Methods:** Human colon cancer HCT116 cells were treated with the isolated compounds of PVW and then subjected to MTT and transwell migration assays.

**Results:** Our results showed that an active compound in PVW, 8,9-didehydro-7-hydroxydolichodial (DHD) inhibited cell viability of HCT116 cells, with IC_50_ value at 6.1 ± 2.2 μM. Interestingly, DHD was not detected in the herbal material of PV. Further investigation revealed that DHD is in fact a heat-generated compound derived from a natural compound present in PV, namely valerosidate. Valerosidate also reduced cell viability in HCT116 cells, with IC_50_ value at 22.2 ± 1.1 μM. Moreover, both DHD (2.75 μM) and valerosidate (10.81 μM) suppressed cell migration in HCT116 cells, with inhibitory rates at 74.8% and 74.6%, respectively. In addition, western blot results showed that DHD (5.5 μM) could significantly increase p53 expression by 34.8% and PTEN expression by 13.9%, while valerosidate (21.6 μM) could increase expressions of p53 and PTEN by 26.1% and 34.6%, respectively in HCT116 cells after 48 h treatment.

**Discussion:** Taken together, this is the first report that a naturally-occurring valerosidate present in PV could actually transform to DHD by thermal hydrolysis, and both compounds exhibited inhibitory effects on cell viability and migration in HCT116 cells via increasing the expressions of tumor suppressors (p53 and PTEN). Our findings demonstrated that valerosidate is present in raw herb PV but not in PVW, while DHD is present in PVW rather than in raw herb PV. This difference in chemical profiles of raw herb and boiled water extract of PV may affect the anti-cancer activity, and hence further investigations are warranted.

## 1 Introduction

*Patrinia villosa* Juss. (PV) as a medicinal herb has been used over thousands of years in China (He et al., 2017). In botany, PV is a perennial herb that typically develops in verdant areas, thickets, or along the edges of forests (Gong et al., 2021). The stems of PV are yellowish green with white coarse anatropous filaments; the leaves are rosulate, long petiolate, ovate, or oblong-lanceolate to ovate-lanceolate (He et al., 2017). The lateral branches of PV are densely hirsute with 5 or 6 pairs, and its corolla is campanulate (He et al., 2017). The flowers are white, which is a special character to distinguish among other *Patrinia* species. As a folk medicine, PV has been used in treating acute appendicitis, hepatitis, tonsillitis, intestinal carbuncle and abscess (Zheng et al., 2012). According to traditional Chinese medicine theory, colorectal cancer is recognized as a carbuncle symptom in the colon and rectum. Thus, PV has been used for treating colorectal cancer by Chinese medicine practitioners for long history as it has been recorded in classical medical books, such as “Tai Ping Sheng Hui Fang”, “Qian Jin Yi Fang”, as well as “Sheng Ji Zong Lu” (Gong et al., 2021). Besides, modern pharmacological studies showed that PV possess potent anti-inflammatory, anti-pathogenic, and anti-cancer activities (He et al., 2017).

In phytochemical studies, over two hundred compounds, mainly flavonoids, organic acids, and iridoids, have been isolated from PV (He et al., 2017; Gong et al., 2021). Among the flavonoids found in PV, such as acacetin, luteolin, apigenin, rutin, quercetin, *etc.* (Gong et al., 2021), they were reported to possess anti-inflammatory, anti-oxidative, and anti-cancer properties (Luo et al., 2022). On the other hand, organic acids are another important group of phytochemicals in PV. More than ten kinds of organic acids have been isolated from PV, such as caffeic acid, ferulic acid, linoleic acid, *etc*. (Gong et al., 2021). These phenolic compounds have anti-oxidative, anti-inflammatory, anti-diabetes, and anti-cancer activities (Rahman et al., 2021). Furthermore, some iridoids found in PV, including valerosidate, patrinoside, villosolside, *etc.* (Gong et al., 2021), have been demonstrated with antiviral, anti-inflammatory, and anti-tumor activities (Wang et al., 2020). Thus, the abundant phytochemicals of PV provide the basis for its pharmacological investigations.

For anti-cancer studies, the ethanol extract of PV has been proven with inhibitory effects on tumor growth in cervical cancer in mice, in which the inhibitory rates were 49.2% and 54.2% at 10 g/kg and 15 g/kg, respectively (Chen et al., 2019). Moreover, saponins isolated from PV suppressed tumor growth in cervical cancer with 35.1% and 57.1% inhibitory rates at the dosages of 50 mg/kg and 100 mg/kg, respectively (Zhang et al., 2008). Patriniaflavanone A isolated from PV also showed a significant inhibitory effect on hepatocarcinoma cells (SMMC-7721) (Zhao et al., 2019). Besides, impecylone A, isolated from PV, was shown to have cytotoxic effects on human liver cancer HepG2 and lung cancer A549 cells (Liu et al., 2019). Nevertheless, up till now, only few preclinical studies on PV related to colon cancer can be found reporting on the inhibitory effects on cancer growth, invasion, and metastasis via regulating PI3K/Akt, and NF-κB signaling pathways, and epithelial to mesenchymal transition (EMT) process (Xia et al., 2018; Li et al., 2023). However, it is important to note that the common practice for consuming PV is boiling it in water but not much scientific evidence can be found on the anti-cancer properties of PV water extract (PVW). In fact, we recently verified the *in vivo* anti-tumor and anti-metastatic activities of PVW in colon cancer mouse models (unpublished data). Therefore, in the present study, we aimed to identify the active component(s) of PVW which exhibit inhibitory activities in colon cancer cells viability and migration. The findings would provide scientific evidences for supporting the clinical use of PVW in colon cancer.

## 2 Materials and methods

### 2.1 General experimental instruments and chemicals

Two different ultra-performance liquid chromatography-mass spectrometers were used: Agilent 1290 Infinity UHPLC system coupled to an Agilent 6530 Accurate-Mass Quadrupole Time-of-Flight Mass Spectrometer with Dual Electrospray Ionization Source (Agilent, CA, United States); and Waters ACQUITY UPLC I-Class PLUS System coupled to a Waters SQ Detector 2 with Electrospray Ionization Source (Waters, MA, United States). The Nuclear Magnetic Resonance (NMR) spectra were performed using a Bruker AVANCE-500 (Bruker Corporation, Germany), and the chemical shifts were referenced to tetramethylsilane (TMS). Column chromatography (CC) was run on silica gel 60 (230–400 mesh, Merck, Germany), and Sephadex LH-20 (GE Healthcare Bio-Sciences AB, Sweden). Thin layer chromatography (TLC) analysis was carried out using Silica gel 60 F_254_ (TLC) plates (Merck, Germany). MS-grade methanol (MeOH) and acetonitrile (ACN) were obtained from RCI Labscan, Ltd. (Bangkok, Thailand). Formic acid was purchased from Thermo scientific, Inc. (Rockford, IL, United States). Ultrapure water was prepared with a Milli-Q system (Millipore, France).

### 2.2 Plant materials

Dried herb of PV (including roots, stems and leaves) was purchased from Zisun Hong Kong Limited (China) and was morphologically identified by Dr. David Tai-Wai Lau of Shiu-Ying Hu Herbarium, The Chinese University of Hong Kong. A voucher specimen (No. 3704) was deposited at the museum of Institute of Chinese Medicine at The Chinese University of Hong Kong.

### 2.3 Herbal extraction and compounds isolation

Dried herb of PV (400 g) were soaked in distilled water at room temperature for 1 h, then boiled at 100°C for 1 h. After cooling, the extract solution was filtered using filter paper, and then added with the same volume of distilled water to extract once more (4 L × 1 h × 2 times). The extract solution was then combined and concentrated using a rotary evaporator to 500 mL under vacuum and lyophilized using a freeze dryer (Labconco, United States) to yield 61.0 g of PVW (15.3% w/w). The dried water extract (20 g) was redissolved in distilled water, then partitioned with n-butanol to yield a butanol extract (PVB, 6 g, 1.5% w/w) and the residual extract.

On the other hand, the dried herb of PV (600 g) were also extracted with 75% ethanol under reflux twice (6 L × 1 h × 2 times) and concentrated *in vacuo* to afford ethanol extract (PVE, 55 g, 9.2% w/w).

#### 2.3.1 Isolation of 8,9-didehydro-7-hydroxydolichodial (DHD)

**FIGURE 1:**
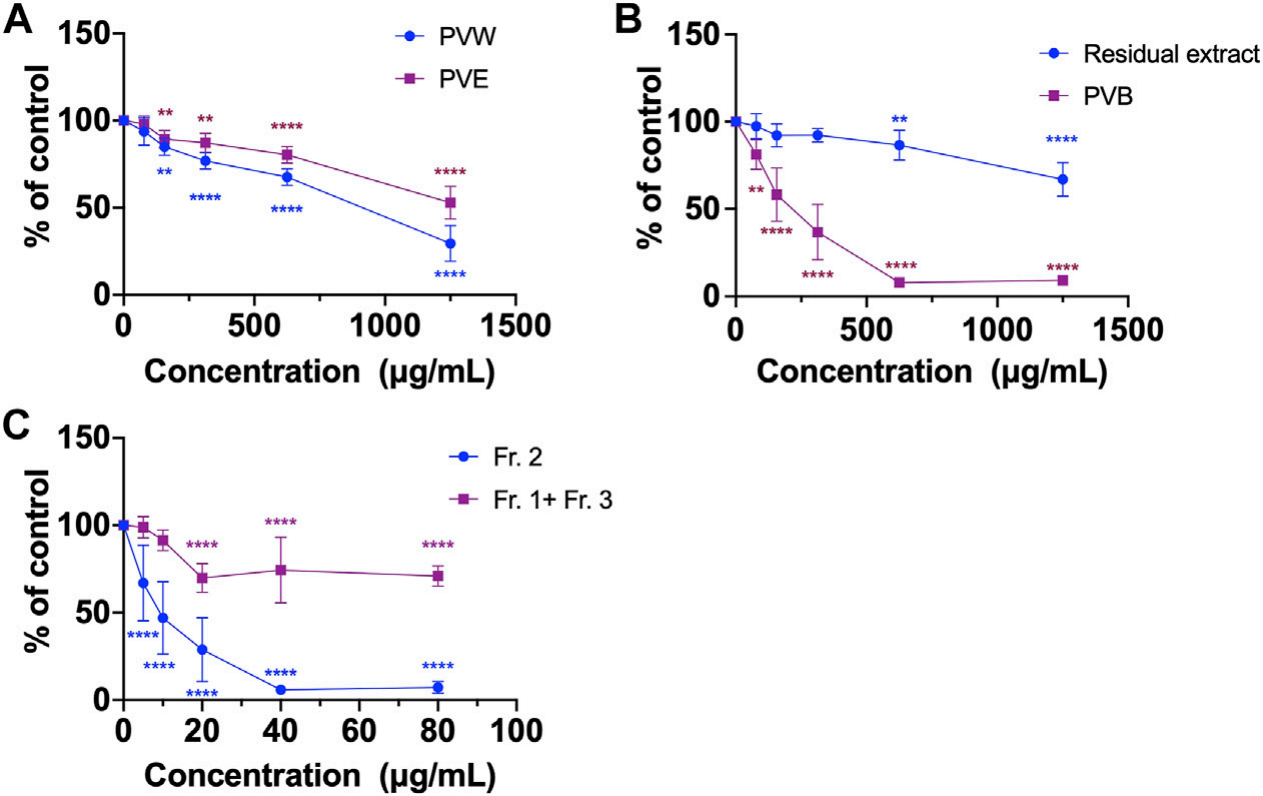
The cell viability of various extracts of PV using MTT assays. **(A)** PVW and PVE; **(B)** PVB and the residual extract; **(C)** Fr. 2 and the combined fraction (Fr. 1 + Fr. 3). All data were presented as mean ± SD, *n* = 3. ^**^ *p* < 0.01, ^****^ *p* < 0.0001 vs. control.

As shown in Supplementary Figure S1 , PVB was subjected to silica gel column chromatography (230–400 mesh) eluted with a gradient of petroleum ether-ethyl acetate (8: 6/4: 6, v/v) to yield three fractions (Fr.1-Fr.3) as analysed by TLC (Supplementary Figure S2). Since the yields of Fr.1 and Fr. 3 were very low, these fractions were combined as one fraction (Fr. 1+ Fr. 3). The effects of fractions (Fr.1 + Fr.3) and Fr. 2 on cell viabilities in HCT116 cells were evaluated. Our MTT results showed that Fr. 2 had better inhibitory effect of cell viability than Fr. 1+ Fr. 3 (Figure 1C). Thus, Fr. 2 (350 mg) was then subjected to a Sephadex LH-20 filtration column eluted with methanol and further purified using another Sephadex LH-20 column eluted with chloroform-methanol (1:1, v/v) to obtain about 48 mg of DHD as identified using NMR spectra. The purity of DHD is >98% as determined by UPLC-MS.

The content of DHD in PVE was determined in order to quantify the amount of DHD present in the raw herb of PV. Since ethanol is a commonly-used solvent to extract naturally occurring components from herbs for chemical analysis (Abubakar and Haque, 2020). Surprisingly, DHD was not detected in PVE. Hence, we speculated that DHD is not naturally present in PV. To prove our hypothesis, distilled water was added into PVE, and then reflux for an hour to obtain the solution. After TLC analysis (Supplementary Figure S3), DHD appeared in the solution. We therefore confirmed that DHD was not a naturally-occurring compound in PV, but it is a heat-processed product generated from PV since no DHD was detected if PV was soaked in water at room temperature (Supplementary Figure S3). So for the next step, we made an attempt to identify the naturally occurring precursor compound which give rise to DHD as detected in PVW.

#### 2.3.2 Extraction and isolation of precursor compound of DHD

As shown in the flow chart of extraction (Supplementary Figure S4), the air-dried herb PV (600 g) were powdered and extracted using 75% ethanol under reflux twice (6 L × 1 h × 2 times). The extracts were combined and concentrated under vacuum condition to give a condensed extract (55 g). The dried residue was resuspended in water and extracted sequentially with petroleum ether, ethyl acetate and butanol, yielding petroleum ether fraction (7 g), ethyl acetate fraction (5 g), butanol fraction (15 g) and the remaining water fraction. We then used a tracking method to check which partition fraction contains the precursor compound of DHD. In brief, about 20 mg of sample from each fraction (petroleum ether fraction, ethyl acetate fraction, butanol fraction and water fraction) were taken and refluxed at 100°C with 2 mL distilled water for 1 h, then allowed to cool at room temperature. The hydrolysates were filtered and analyzed by TLC. After reflux and TLC detection, we could easily detect which fraction contained the precursor of DHD. All these fractions were labeled as positive fractions in Supplementary Figure S4. The butanol fraction, which was found positive, was resuspended in water and then passed through a D101 resin column eluted with methanol-water (0:1–1:0) to afford three fractions (Fr. A- Fr. C). After tracking analysis, Fr. B was found positive. Fr. B was then passed through a Sephadex LH-20 column with a methanol eluent to give four fractions (Fr. B1- Fr. B4). Similarly, Fr. B2, which was found positive, was then subjected to silica gel column chromatography (230–400 mesh) and eluted with a gradient of ethyl acetate-methanol-water (15:1:1 → 5:1:1, v/v) to give three subfractions (Fr. B2-1- Fr. B2-3). Finally, Fr. B2-2, which was found positive, was subjected to Sephadex LH-20 column eluted with acetone and further purified using another Sephadex LH-20 column eluted with methanol to yield about 60 mg of compound, which was later identified as valerosidate using NMR spectra, with purity >98% as determined by UPLC-MS (as shown below).

### 2.4 Ultra performance liquid chromatography-quadrupole time of flight (UPLC-QTOF) mass spectrometer analysis

UPLC-QTOF analysis was used for qualitatively detecting DHD and valerosidate in the extract of PV. The analysis was conducted using an Agilent 1290 UHPLC coupled to 6530 Accurate-Mass Quadrupole Time-of-Flight Mass Spectrometer with Dual Electrospray Ionization Source (CA, United States). The column used was Agilent ZORBAX Eclipse Plus C_18_ RRHD, 1.8 µm, 2.1 mm × 50 mm with guard column. The chromatographic separation was conducted at 30°C under gradient conditions at a flow rate of 0.4 mL/min. Mobile phase: (A) 0.1% formic acid in deionized water; and (B) 0.1% formic acid in acetonitrile, with gradient: 0–2 min, 5% B; 2–2.5 min, 5%–13% B; 2.5–9 min 13% B; 9–10 min 13%–100% B. The column was flushed with 100% mobile phase B for 2 min and re-equilibrium for another 2.5 min after each injection. Data were collected between 0–9 min. As the curtain and collision gas, 10 L/min of high-purity nitrogen was utilized. The temperature of the dehydrating gas was set to 350°C, and the pressure of the nebulizer was set to 35 psi. At a discharge voltage of 4000 V, spectra were captured in negative ion mode. The mass scan range was between 100–1,000 m/z. Agilent MassHunter Workstation Qualitative Analysis Software (CA, United States, version B.07.00) was used to analyze the data. Valerosidate was determined at 485.1998 m/z [M + Na]^+^ and DHD was determined at 163.0754 m/z [M-H_2_O + H]^+^.

### 2.5 Ultra performance liquid chromatography-mass spectrometer (UPLC-MS) analysis

UPLC-MS was used for quantitative analysis of DHD and valerosidate present in the extract of PV. The equipment used was a Waters ACQUITY UPLC I-Class PLUS System consisting of a binary high-pressure pump with vacuum degasser, a single quadrupole mass detector (SQD2), an autosampler and the Acquity UPLC^®^ BEH C_18_ column (2.1 mm × 50 mm, 1.7 μm; Waters, MA, United States). Gradient elution was utilized in the chromatographic separation method under the following conditions: column temperature, 30°C; injection volume, 1 μL; flow rate, 0.5 mL/min. Mobile phases A and B were 0.1% v/v formic acid (FA) in water and 0.1% v/v FA in acetonitrile, respectively. Elution in UPLC used the following linear gradient: 0–2 min, 5% B; 2–2.5 min, 5%–13% B; 2.5–6.5 min, 13% B; 6.5–7 min, 13%–100% B; 7–9 min, 100% B; and 9–11 min, 5% B. The mass spectrometric parameters were set as below: the ion source was electrospray ionization (ESI); the mass spectra were recorded in positive mode; nitrogen nebulizing gas was used for desolvation at a flow rate of 700 L h^−1^ at 400°C; the capillary voltage was 3000 V; the temperature of the ionization source was 150°C; and the cone voltage was 50 V. Data were acquired in single ion recording (SIR) mode with unit resolution. Data were generated and analyzed on the Masslynx v 4.2 software.

### 2.6 Hemolytic assay

Hemolytic activity was determined using mouse red blood cells with serial concentrations of each tested compounds. Whole blood was collected from Balb/c mice under anesthesia and the mice were sacrificed by cervical dislocation as described in the previous study (Sæbø et al., 2023). The animal experiments were approved by the Animal Experimentation Ethics Committee of CUHK (Ref no. 21-287-MIS). Red blood cells were collected after centrifugation of whole blood. Red blood cells (1 mL) were diluted into 9 mL of PBS. Then, 100 μL of diluted red blood cells were added into a 96-well plate with same volume of serial dosages of tested compounds. 1% triton-X100 solution was used as positive control. After 2 h incubation at 37°C with 5% CO_2_ in an incubator (BINDER, Germany), the 96-well plate was centrifuged at 2000 × *g* for 5 min. Absorbance of the supernatant was measured at 540 nm using a μQuant microplate spectrophotometer (Biotek, United States). The percentage of hemolysis was calculated using the formula as below:

$$Hemolytic\;activity=\frac{O.D.\;sample-O.D.\;blank\;control}{O.D.\;positive\;control-O.D.\;blank\;control}\times 100\;\%$$

Where O.D. is optical density.

### 2.7 Cell culture

Human colon cells HCT116 and the human fibroblast cells Hs27 were purchased from ATCC (American Type Culture Collection, United States). HCT116 cells were grown in McCoy’s 5A medium supplemented 10% (v/v) fetal bovine serum (FBS) and 1% (v/v) penicillin/streptomycin. Hs27 cells were grown in Dulbecco’s Modified Eagle’s medium (DMEM) supplemented with 10% (v/v) fetal bovine serum (FBS) and 1% (v/v) penicillin/streptomycin. All cell culture media and supplements were obtained from Thermo Fisher Scientific (Waltham, MA United States). All cells were incubated in an incubator (BINDER, Germany) at 37°C and 5% CO_2_.

### 2.8 MTT assay

The cell viability of colon cancer cells and normal cells after incubating with crude extracts of PV or isolated compounds were tested using 3-(4,5-dimethylthiazol-2-yl)-2,5-diphenyltetrazolium bromide (MTT) assay. Briefly, 5×10^3^ cells per well were seeded and incubated overnight in a 96-well microplate. The cells were then incubated for 48 h with 100 μL of PV extracts or compounds at indicated concentrations. Then, MTT solution (30 μL of 5 mg/mL) was added to each well, and the cells were incubated for an additional 4 h. MTT crystals were dissolved in dimethyl sulfoxide, and the absorbance at 540 nm (OD_540_) was measured using a μQuant microplate spectrophotometer (Biotek, United States). The half maximal cytotoxic concentration (CC_50_) was the concentration of a compound that can kill 50% of the cell viability on normal cells (Hs27 in the present study). The half maximal inhibitory concentration (IC_50_) is the concentration of a compound that can inhibit 50% of the cell viability on cancer cells (HCT116 in the present study). The CC_50_ and IC_50_ were determined by plotting a dose-response curve, where the concentration of the compound was plotted against the percentage of surviving cells. To calculate the CC_50_ and IC_50_ value, the dose-response curve was analyzed to determine the concentration of the compound that produces a 50% reduction in cell viability. This is typically performed using a mathematical model such as a sigmoidal dose-response curve or a linear regression analysis (Zhao et al., 2015). The cell viability of compounds was calculated by the formula:

$$Cell\;viability\;\left(\%\right)=\frac{Sample\;absorbance-Blank\;control\;absorbance}{Control\;absorbance-Blank\;control\;absorbance}\times 100\;\%$$

Then, the cell viability (*y*-axis) was plotted against the log drug concentration using GraphPad Prism v 8.0 software, and the dose-response curve was obtained to determine the CC_50_ or IC_50_ value. The selectivity index (SI) was calculated, with formula SI = 50% cytotoxic concentration (CC_50_)/50% inhibitory concentration (IC_50_).

### 2.9 BrdU cell proliferation assay

The cell proliferation was determined using the 5-bromo-2′-deoxyuridine (BrdU, Roche, IN, United States) assay according to the manufacturer’s instructions. Briefly, 5 × 10^3^ cells per well were seeded and incubated overnight in a 96-well microplate. The cells were then exposed to 100 μL of the indicated concentrations of PV extracts or compounds, for 48 h before being labeled with BrdU for an additional 4 h. The absorbance was measured using a microplate spectrophotometer (μQuant, Biotek, CA, United States) after washing, fixing, and reacting cells with anti-BrdU reagent.

### 2.10 Transwell migration assay

The cell migration on colon cancer cells was evaluated using transwell migration method. In brief, 100 μL of 5×10^4^ cells in serum-free medium with or without tested compounds were added to the upper chamber of a transwell (Corning, NY, United States), while 500 μL of a chemoattractant medium containing 10% FBS was added to the lower chamber. After 24 h of incubation, the cells were fixated for 3 min with methanol and stained for 5 min with hematoxylin. The cells on the top surface of the membrane were removed using a cotton swab, and the residual adherent cells were photographed using an Olympus IX-71 microscope equipped with a digital camera (Olympus, Tokyo, Japan). Using Image J software, the number of migrated cells was determined, which reflected their ability to migrate.

### 2.11 Western blot assay

HCT116 cells were inoculated overnight in 6-well plates. The cells were then exposed to test compounds for 48 h. After that, the cells were lysed on ice with a lysis buffer (Beyotime, Shanghai, China). The concentration of protein was measured using a BCA kit (Thermo Scientific, MA, United States). 30–50 μg of protein were deposited onto 10% SDS-PAGE gels before being transferred to polyvinylidene fluoride (PVDF) membranes. An hour was spent blocking membranes with 5% non-fat milk in Tris-buffered saline Tween 20 (TBST). The blots were incubated overnight at 4°C with primary antibodies (1:1,000), including rabbit antibodies p53 and PTEN (1:1000, Cell Signaling Technology, MA, United States), and mouse antibodies β-actin (1:3000, Merck, MO, United States) as listed in Supplementary Table S2. After three 15 min washes with TBST solution, the membranes were incubated for 1 h with secondary horseradish peroxidase-conjugated anti-mouse or anti-rabbit antibodies (1:3,000, Thermo Scientific, MA, United States). Blots were then visualized using the ChemiDoc XRS + Imaging System (Bio-Rad, CA, United States) after the ECL reagent detection (GE Healthcare Life Sciences, Sweden). The bands were measured using Image J (NIH, United States). Each protein sample’s band intensities were normalized to its own internal standard proteins (β-actin). The quantitative results were presented as a multiple of the untreated control.

### 2.12 Statistical analysis

Data from the cell culture experiments were expressed as mean ± standard deviation (S.D.). Differences among groups were tested using one-way ANOVA, followed by *post-hoc* Dunnett’s multiple comparison test, *p* values < 0.05 were considered statistically significant. Statistical analysis was performed using GraphPad Prism v 8.0 software (La Jolla, United States).

## 3 Results and discussion

Most of the Chinese herbal medicines are traditionally used as decoction, and the common practice for consuming herbal medicines is boiling them in water. However, in modern pharmacological studies, it is common to study herbal extracts prepared with different organic solvents (such as methanol and ethanol) in order to extract the active compounds (Zhao et al., 2015). This applies to the case of PV. PV ethanol extract has been demonstrated to possess inhibitory effects on cervical and colon tumor growth in mice (Chen et al., 2019; Li et al., 2023). However, when PV was prescribed for colon cancer patients, they would take the aqueous decoction (i.e., water extract) orally (McCulloch et al., 2016). Hence, we focused on investigation of the water extract of PV and its active compounds in colon cancer. Herein, we first compared the inhibitory activities of cells viability between PVW and PVE in HCT116 cells using MTT assay. The MTT assay is a popular and versatile method for assessing cells viability, involving the conversion of MTT dye to an insoluble formazan by mitochondrial reductase (Kumar et al., 2018). Results showed that the cell viabilities of HCT116 cells were reduced after treating with PVW and PVE for 48 h. The IC_50_ value of PVW was 806.0 ± 8.7 μg/mL, which was lower than that of PVE (IC_50_ > 1,250 μg/mL) in HCT116 cells (Figure 1A), indicating that the active compound(s) were present in PVW.

**FIGURE 2:**
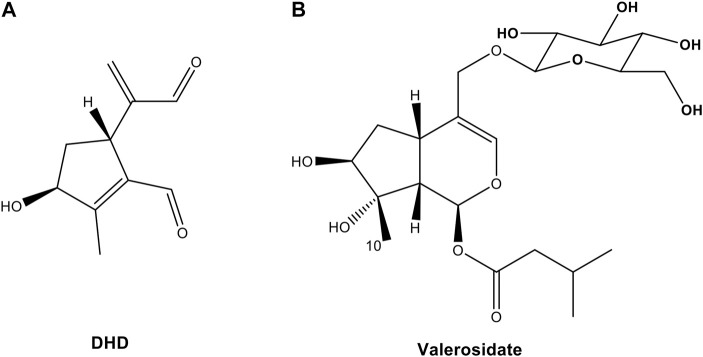
Chemical structures of **(A)** 8,9-didehydro-7-hydroxydolichodial (DHD) and **(B)** valerosidate from PV.

In order to find out the active compounds, PVW was partitioned with n-butanol to obtain n-butanol part of PVW (PVB) and residual extract. The effects of PVB and residual extract on cell viability was compared using MTT assay. Results showed that PVB (IC_50_ at 198.4 ± 10.9 μg/mL) exhibited stronger inhibitory activity on cell viability in HCT116 cells than residual extract (IC_50_ > 1,250 μg/mL) (Figure 1B), indicating the active compound(s) would be existed in PVB. Next, the active compounds of PVB were further explored by separating PVB into three fractions (Fr.1-Fr. 3, Fig. S1 and S2) via silica gel column chromatography eluted by petroleum ether-ethyl acetate. Results showed that the inhibitory effect on cell viability of Fr. 2 (IC_50_ at 8.95 ± 1.3 μg/mL) was much stronger than that of the combined fraction (Fr. 1 + Fr .3, IC_50_ > 80 μg/mL) in HCT116 cells (Figure 1C), suggesting that active compound(s) should be present in Fr. 2. So, we further purified Fr. 2 to obtain a compound, which was eventually identified as DHD by NMR analysis. The chemical characteristics of DHD were described as below:

DHD (Figure 2A) was isolated as a light yellow oil with a molecular formula of C_10_H_12_O_3_ based on HR-ESI-MS at m/z 163.0754 [M- H_2_O + H]^+^ (calcd 163.0754). ^1^H-NMR (Methanol-*d*_4_, 500 MHz) *δ*_H_ 9.93 (1H, s, H-1), 9.48 (1H, s, H-3), 5.94 (2H, d, *J* = 1.8 Hz, H-11), 3.92 (1H, dd, *J* = 9.0, 1.2 Hz, H-5), 4.71 (1H, m, H-7), 2.20 (3H, s, H-10), 1.95 (1H, m, H-6a), 1.89 (1H, m, H-6b). ^13^C{^1^H}-NMR (Methanol-*d*_4_, 125 MHz) *δ*_C_ 195.7 (C-3), 190.3 (C-1), 166.7 (C-8), 152.8 (C-4), 138.8 (C-9), 133.9 (C-11), 80.0 (C-7), 40.6 (C-5), 40.3 (C-6), 11.6 (C-10). The compound was identified by ^1^H and ^13^C-NMR analysis (Supplementary Figures S5, S6) in comparison to the reference (Quan et al., 2020), and the structure was further confirmed by ^1^H-^1^H COSY, HSQC, HMBC, and ROESY data (Supplementary Figures S7–S10).

**FIGURE 3:**
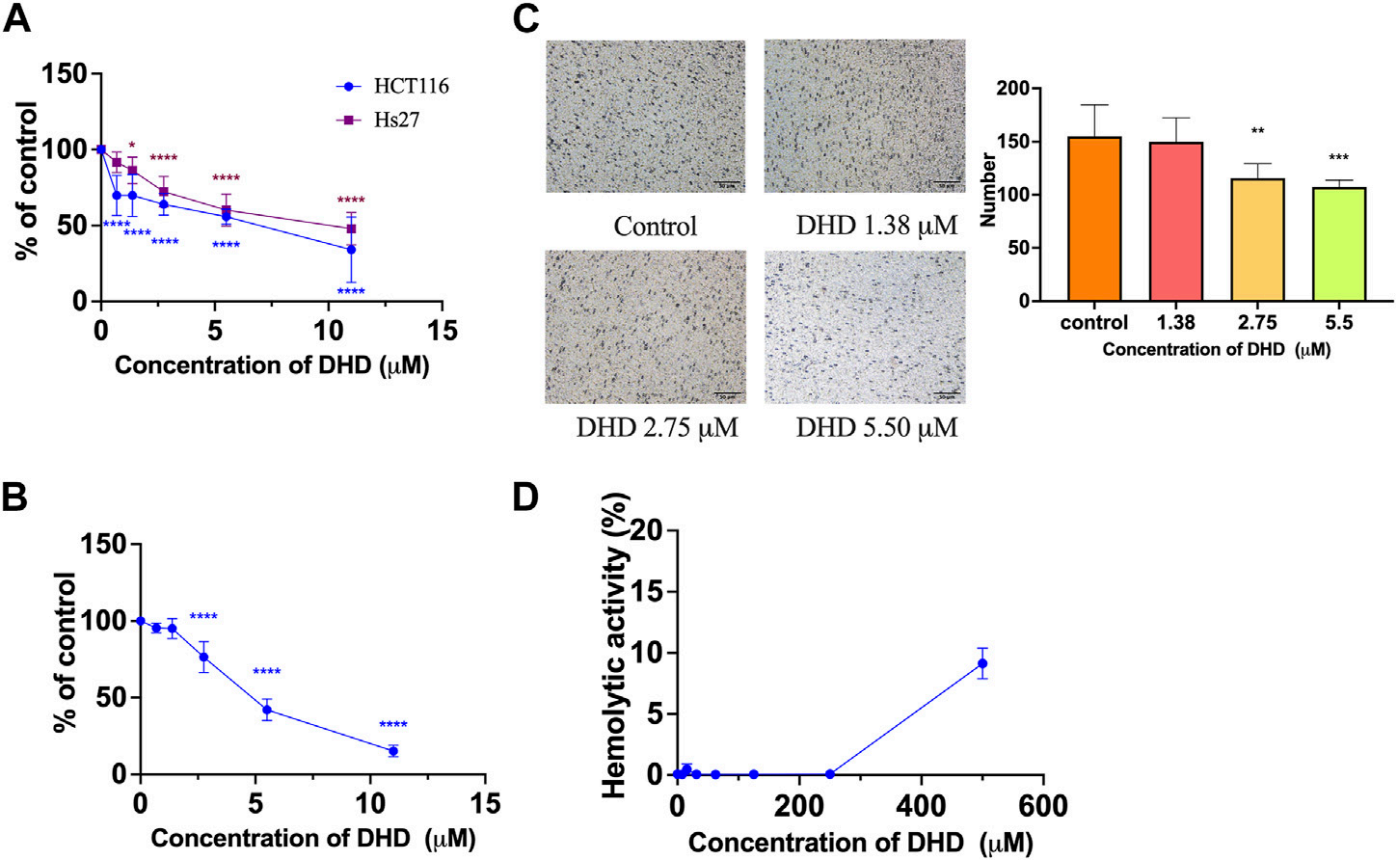
The cell viability, anti-proliferative and anti-migratory effects of DHD in human colon cancer HCT116 cells. **(A)** The cell viability of DHD in HCT116 and human fibroblast Hs27 cells; **(B)** The anti-proliferative effect of DHD in HCT116 cells; **(C)** The anti-migratory effect of DHD in HCT116 cells. Micro-photos showed the migrated cells on the transwell membrane (scale bar: 50 μm). Histogram representatives the number of the migrated cancer cells. **(D)** Hemolytic activity of DHD. All data were presented as mean ± SD, *n* = 3. ^*^ *p* < 0.05, ^**^ *p* < 0.01, ^***^ *p* < 0.001, ^****^ *p* < 0.0001 vs. control.

Regarding the biological activity of DHD, only a previous study reported that DHD isolated from *Valeriana jatamansi* exhibited cytotoxic effect against human glioma stem cells (Quan et al., 2020), however, there was no report on colon cancer cells. Thus, the effect of DHD on cell viability was evaluated by MTT assay in colon cancer cells HCT116 in the present study. Results showed that DHD possessed suppressive effect on cell viability in HCT116 cells with IC_50_ value at 6.1 ± 2.2 μM after 48 h treatment (Figure 3A). DHD also exhibited suppressive effect on cell viability in normal human fibroblast cells (Hs27) with IC_50_ value at 9.4 ± 4.4 μM after 48 h treatment, and the SI index of DHD was 1.54, indicating the selective cell viability of DHD was inferior (Figure 3A). Furthermore, the anti-proliferative activity of DHD was assessed by BrdU cell proliferation assay. The assay involves the incorporation of a synthetic nucleoside analog (bromodeoxyuridine, BrdU) into replicating DNA of the cells, which can be detected using anti-BrdU antibody to assess cell proliferation, differentiation, or DNA repair (Matatall et al., 2018). Results showed that DHD at more than 2.75 µM could significantly inhibit 76.4% proliferation of HCT116 cells (*p* < 0.0001) (Figure 3B). Furthermore, the cell migration of HCT116 cells was evaluated by transwell migration assay. Transwell migration assay is an *in vitro* technique widely used in cancer research to measure the migration potential of cancer cells to evaluate the effect of drugs on cancer cells migration (Oner and Kobold, 2022). As shown in Figure 3C, DHD at 2.75 μM could significantly inhibit cell migration in HCT116 cells with inhibitory rate of 74.8% (*p* < 0.01). Besides, DHD at 500 µM showed hemolytic activity (9.12% ± 1.25%, Figure 3D), indicating DHD has slight toxicity towards erythrocytes. Thus, DHD could suppress cells viability, proliferation and migration in HCT116 cells.

**FIGURE 4:**
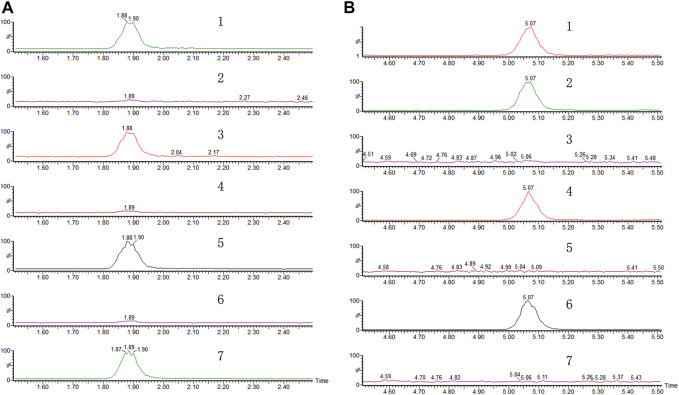
Representative chromatograms of **(A)** DHD and **(B)** valerosidate. (1) Referenced standard; (2) water extract of PV by maceration. (3) Refluxed extract of (2) in water. (4) Extract of PV by reflux in 75% ethanol. (5) Refluxed extract of (4) in water. (6) Extract of PV by maceration in 75% ethanol. (7) Refluxed extract of (6) in water.

Since DHD was shown to be an active compound in PVW, we then tried to measure its content in the PV raw herb. Since ethanol is a commonly used solvent to extract naturally occurring components from herbal medicines in chemical analysis (Abubakar and Haque, 2020), we tried to quantify DHD in PVE. Surprisingly, from our results, DHD could not be detected in PVE, whereas it was shown to be present in the water reflux solution of PVE (Figure 4). Similarly, this phenomenon also occurred in PVW prepared by maceration (herb material soaked in distilled water at room temperature overnight) and reflux method, which has been demonstrated by UPLC (Figure 4A) and TLC (Supplementary Figure S3) analysis. So, we suspected that DHD is not a naturally-occurring compound in PV, but it might be a heat-hydrolyzed product from a naturally-occurring compound in PV. We then attempted to identify the naturally-occurring precursor compound which gives rise to DHD in PVW (i.e., after heating PV in water). Based on this hypothesis, our research group designed the experimental plan to trace the precursor compound of DHD (Supplementary Figure S4) and managed to isolate a compound from PVE which might be the precursor compound of DHD. This compound was identified as valerosidate by NMR analysis and by comparison to the reference (Inouye et al., 1974), with the chemical characteristics described as below:

Valerosidate (Figure 2B) was isolated as a light yellow oil with a molecular formula of C_21_H_34_O_11_ based on HR-ESI-MS at m/z 485.1998 [M + Na]^+^ (calcd 485.1993). ^1^H-NMR (Methanol-*d*_4_, 500 MHz) *δ*_H_ 6.12 (1H, d, *J* = 3.9 Hz, H-1), 6.28 (1H, br s, H-3), 2.99 (1H, m, H-5), 1.94 (2H, m, H-6), 3.70 (1H, t, *J* = 3.6 Hz, H-7), 2.20 (1H, dd, *J* = 10.3, 3.9 Hz, H-9), 1.31 (3H, s, H-10), 4.04 (1H, d, *J* = 11.4 Hz, H-11a), 4.21(1H, d, *J* = 11.4 Hz, H-11b), 2.16 (2H, d, *J* = 7.2 Hz, H-2′), 2.04 (1H, m, H-3′), 0.92 (6H, d, J = 6.7 Hz, H-4′, H-5′), 4.25 (1H, d, *J* = 7.8 Hz, H-1″), 3.14 (1H, dd, *J* = 7.8, 1.7 Hz, H-2″), 3.31 (1H, m, H-3″), 3.21 (1H, dd, *J* = 4.0, 2.2 Hz, H-4″), 3.22 (1H, m, H-5″), 3.61 (1H, m, H-6″a), 3.82 (1H, dd, *J* = 11.9, 1.9 Hz, H-6″b). ^13^C{^1^H}-NMR (Methanol-*d*_4_, 125 MHz) *δ*_C_ 91.9 (C-1), 139.5 (C-3), 117.0 (C-4), 32.1 (C-5), 38.1 (C-6), 80.9 (C-7), 82.3 (C-8), 48.3 (C-9), 22.9 (C-10), 70.0 (C-11), 173.2 (C-1′), 44.3 (C-2′), 26.8 (C-3′), 22.7 (C-4′, C-5′), 103.6 (C-1″), 75.2 (C-2″), 78.1 (C-3″), 71.7 (C-4″), 77.9 (C-5″), 62.8 (C-6″). The structure and stereochemical assignments of this natural iridoid glycoside were also confirmed by HMBC (Fig. S11) and ROESY (Fig. S12).

**FIGURE 5:**
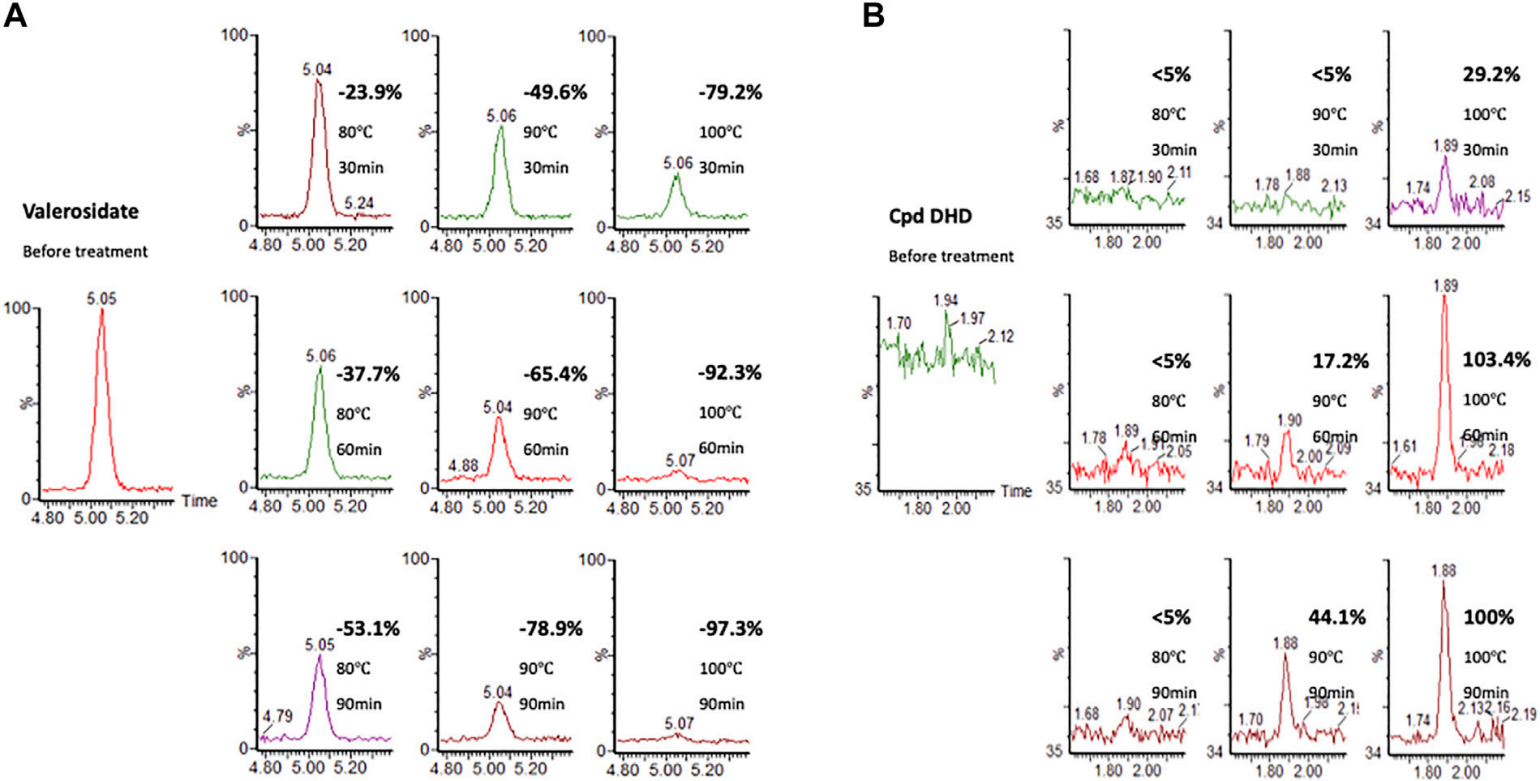
The heat denaturation of a purified valerosidate under different heating temperatures and time. **(A)** Valerosidate (% is calculated by comparing the amount of valerosidate without heating). **(B)** DHD (% is calculated by comparing the amount of DHD with 100°C heating for 90 min).

Our UPLC-MS results showed that valerosidate was only detected in either maceration method (i.e., PV raw herb soaked in distilled water at room temperature (about 25°C overnight) or refluxed in 75% ethanol extract, which is at low heat (below 80 °C) condition (Figure 4B). In addition, we found that valerosidate is actually thermally unstable and it decomposes starting from 80°C of heating. Its decomposition is completed close to 1 h of 100°C heating (Figure 5A). This can explain why valerosidate is not found in the traditional decoction of PV since it has been subjected to boiling for at least 1 h in water. In order to further prove that DHD is a heat-generated product of the pure compound valerosidate by thermal hydrolysis, we tried to detect if DHD appeared after the heat denaturation. As shown in Figure 5B, DHD was formed with the decomposition of valerosidate, and also reached its peak production at 1 h of heating at 100°C. This provided direct evidence that DHD is a decomposition product of valerosidate. Besides, the contents of valerosidate and DHD were detected in PV water extract prepared by maceration and reflux method, respectively (Figure 5B). Results showed that DHD was only detected in reflux-extracted PVW, while valerosidate was only detected in maceration-extracted PVW (Supplementary Table S1). Taken together, our findings confirmed that DHD is a heat-generated product of valerosidate in PV.

**FIGURE 6:**
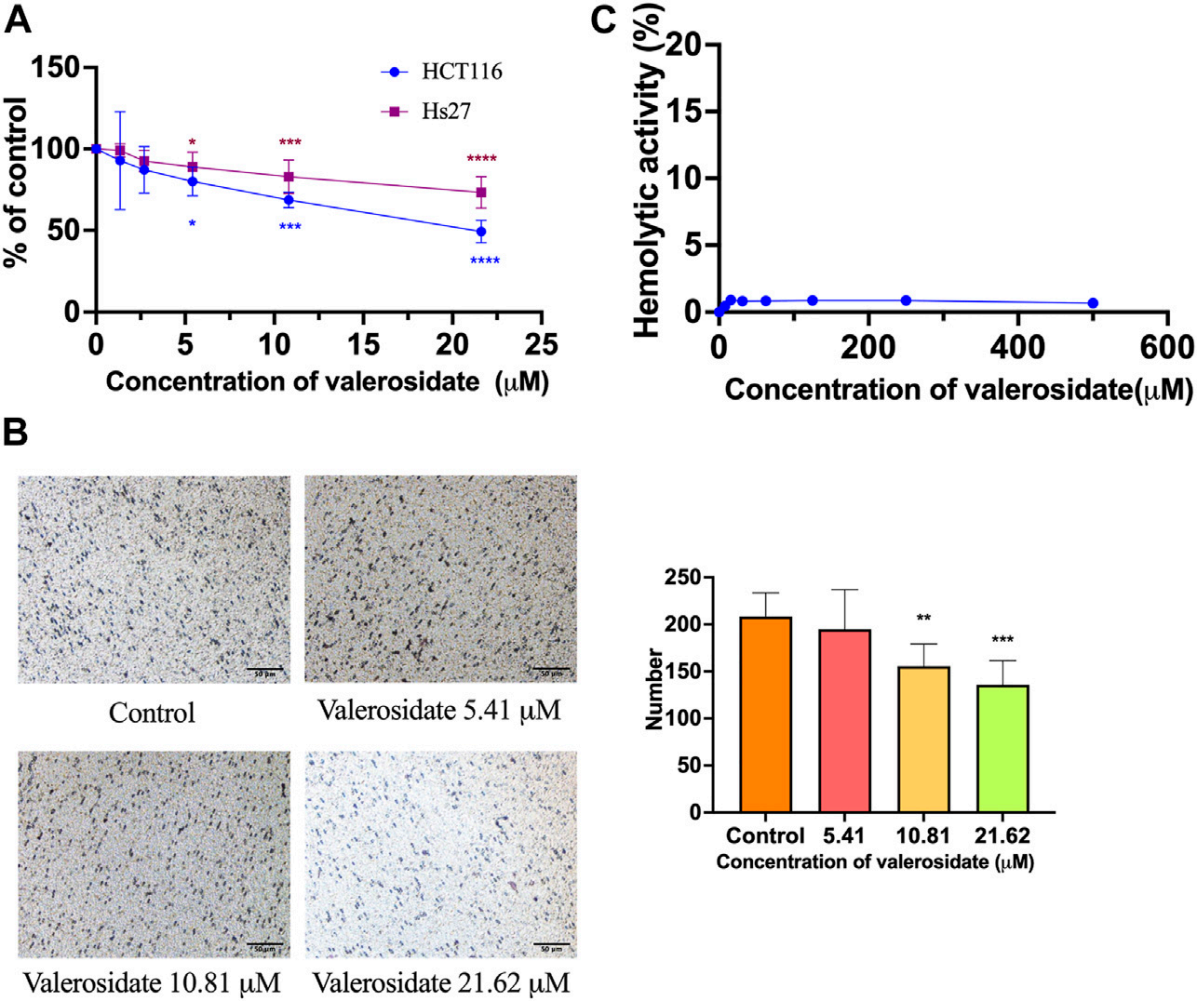
*In vitro* results of the cell viability, anti-migration, and hemolytic activity of valerosidate. **(A)** The cytotoxic effect of valerosidate in HCT116 and Hs27 cells; **(B)** The anti-migratory effect of valerosidate in HCT116 cells. Micro-photos showed the migrated cells on the transwell membrane (scale bar: 50 μm). Histogram representatives the number of the migrated cancer cells. **(C)** Hemolytic activity of valerosidate. All data were presented as mean ± SD, *n* = 3. ^*^ *p* < 0.05, ^**^ *p* < 0.01, ^***^ *p* < 0.001, ^****^ *p* < 0.0001 vs. control.

In fact, valerosidate has been previously reported as a naturally-occurring compound in PV (Lee et al., 2013), but the anti-cancer activity of valerosidate is yet to be explored. In this study, MTT results showed that valerosidate exhibited inhibitory effect on cell viability of HCT116 cells, with IC_50_ value of 22.2 ± 1.1 μM for 48 h treatment, while it exhibited mild inhibitory activity on cell viability in Hs27 cells (IC_50_ value = 71.3 ± 1.1 μM). The SI index was 3.21, which was higher than that of DHD, indicating valerosidate has selective inhibitory activities in cancer cells than DHD (Figure 6A). Transwell migration assay results showed that valerosidate at 10.8 μM could significantly inhibit cell migration in HCT116 cells with inhibitory rate was 74.58% (*p* < 0.01) (Figure 6B). Besides, valerosidate at dosages of 0–500 μM did not show any hemolytic activity on mouse red blood cells, indicating it has less toxicity than DHD (Figure 6C). Hemolytic assay is commonly used to evaluate the potential for hemolysis-induced toxicity of chemical compounds, particular in drug development (Sæbø et al., 2023). Hemolysis, the destruction of red blood cells, can lead to severe health complications, including anemia (Sæbø et al., 2023). Therefore, hemolytic assay is a crucial tool in drug development for determining the safety and pharmacological activity of drug candidates.

**FIGURE 7:**
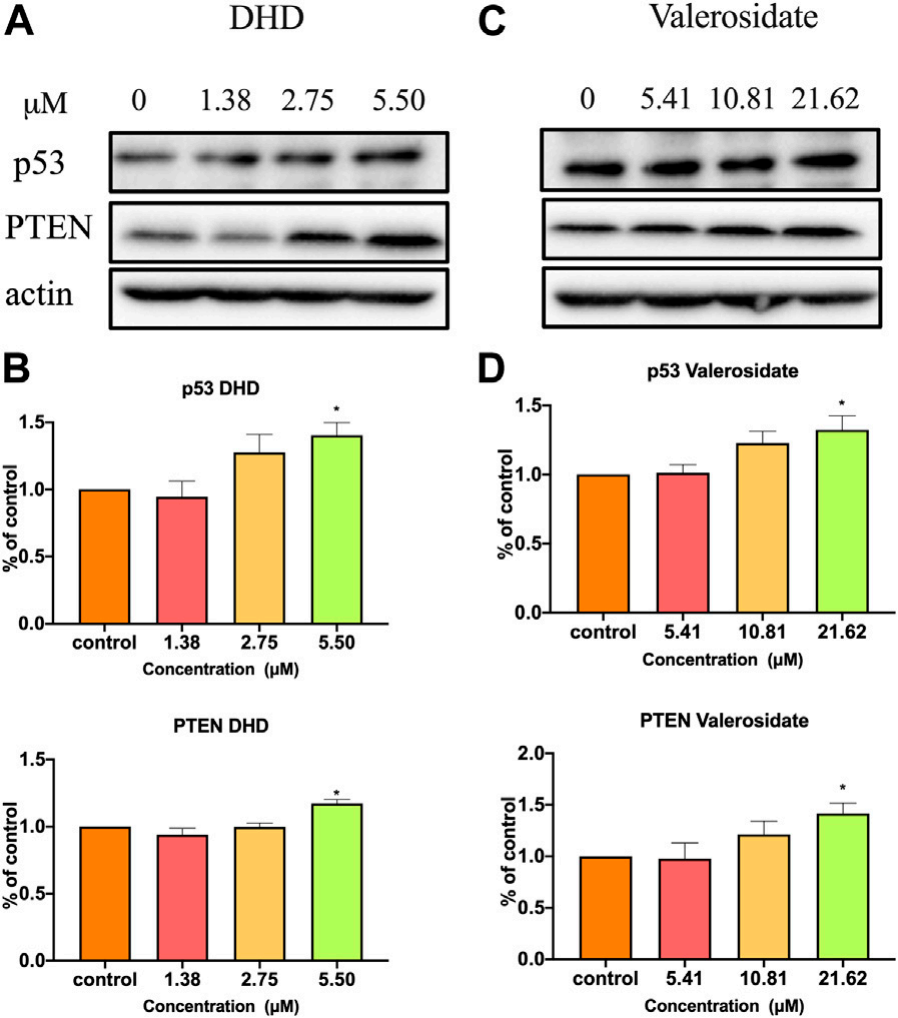
The effects of DHD and valerosidate on protein expressions of p53 and PTEN in HCT116 cells. Representative blots showed the effects of **(A)** DHD and **(C)** valerosidate on the expressions of p53 and PTEN proteins in HCT116 cells. **(B,D)** Histograms showed the quantitative results of the expressions of target proteins which were normalized with corresponding β-actin expressions and expressed as percentage of control. All data were presented as mean + SD, *n* = 3. ^*^ *p* < 0.05 vs. control.

Since both DHD and valerosidate exhibited cell viability and anti-migratory effects on HCT116 cells, their regulatory effects on cancer-related proteins expressions were further explored. P53 and PTEN are crucial tumor suppressors in colorectal cancer (Uboveja et al., 2022). In other words, the loss of p53 or PTEN contributes to aggressive tumor growth and migration (Liebl and Hofmann, 2021; Serebriiskii et al., 2022). Our Western blot results showed that DHD at 5.5 μM could significantly increase p53 expression by 34.8% and PTEN expression by 13.9% (Figures 7A, B), while valerosidate at 21.6 μM could significantly increase p53 expression by 26.1% and PTEN expression by 34.6% in HCT116 cells after 48 h treatment (Figures 7C, D), as compared to vehicle control. Therefore, the inhibitory effects of both DHD and valerosidate on cell viability and migration might be related to the increase of p53 and PTEN expressions.

In order to develop DHD or valerosidate as anti-cancer agents for colon cancer, further preclinical experiments and clinical trials need to be conducted. For example, tumor-bearing animal studies will be required to confirm the efficacy of both compounds in colon cancer. Besides, the pharmacokinetic study and drug safety of these two compounds should also be performed and evaluated.

## 4 Conclusion

This study has proven that DHD in PVW is in fact a heat-generated product of valerosidate present in the raw herb PV. This is the first report that a naturally-occurring valerosidate present in PV could actually transform to DHD by thermal hydrolysis, and both compounds exhibited potent inhibitory effects on cells viability and migration in colon cancer HCT116 cells. Besides, both DHD and valerosidate could increase the expressions of tumor suppressor proteins p53 and PTEN. Our findings demonstrated that different ways of preparing the water extract of PV (by maceration or boiling) may result in differences in the chemical constituents, which may in turn affect the anti-cancer activity, and hence further investigations are warranted. This study raises an important point for further consideration: how the raw herb PV should be prepared for patients in order to achieve better anti-cancer effect in colon cancer, pending further clinical investigations.

## Data Availability

The original contributions presented in the study are included in the article/Supplementary Material, further inquiries can be directed to the corresponding author.

## References

[B1] Abubakar A. R. Haque M. (2020). Preparation of medicinal plants: Basic extraction and fractionation procedures for experimental purposes. J. Pharm. Bioallied Sci. 12 (1), 1–10. 10.4103/jpbs.jpbs_175_19 32801594 PMC7398001

[B2] Chen L. Chen P. Zhang T. Tian L. M. (2019). The study on anti-tumor effect and its mechanism on sarcoma U14 mice of cervical cancer by the alcohol extract of *Patrinia villosa* Juss. Heilongjiang Med. Pharm. 42 (3), 63–65.

[B3] Gong L. Zou W. Zheng K. Shi B. Liu M. (2021). The herba patriniae (caprifoliaceae): A review on traditional uses, phytochemistry, pharmacology and quality control. J. Ethnopharmacol. 265, 113264. 10.1016/j.jep.2020.113264 32846192 PMC7443212

[B4] He X. Luan F. Zhao Z. Ning N. Li M. Jin L. (2017). The genus *Patrinia*: A review of traditional uses, phytochemical and pharmacological studies. Am. J. Chin. Med. 45 (4), 637–666. 10.1142/s0192415x17500379 28595500

[B5] Inouye H. Ueda S. Uesato S. Shingu T. Thies P. (1974). Die absolute konfiguration von valerosidatum und von didrovaltratum. Tetrahedron 30 (15), 4082–2325. 10.1016/s0040-4020(01)97387-9

[B14] Kumar P. Nagarajan A. Uchil P. D. (2018). Analysis of cell viability by the MTT assay, Cold Spring Harb. Protoc. 2018 (6), pdb.prot095505–471. 10.1101/pdb.prot095505 29858338

[B6] Lee J. Y. Kim J. S. Kim Y. S. Kang S. S. (2013). Glycosides from the aerial parts of *Patrinia villosa* . Chem. Pharm. Bull. (Tokyo) 61 (9), 971–978. 10.1248/cpb.c13-00306 23800854

[B7] Li X. C. Wang S. Yang X. X. Li T. J. Gu J. X. Zhao L. (2023). *Patrinia villosa* treat colorectal cancer by activating PI3K/Akt signaling pathway. J. Ethnopharmacol. 309 (1), 116264. 10.1016/j.jep.2023.116264 36868440

[B8] Liebl M. C. Hofmann T. G. (2021). The role of p53 signaling in colorectal cancer. Cancers (Basel) 13 (9), 2125. 10.3390/cancers13092125 33924934 PMC8125348

[B9] Liu Y. Liu W. Chen C. Xiang Z. Liu H. (2019). A Cytotoxic natural product from *Patrinia villosa* Juss. Anticancer Agents Med. Chem. 19 (11), 1399–1404. 10.2174/1871520619666190416101014 31038075

[B10] Luo Y. Jian Y. Liu Y. Jiang S. Muhammad D. Wang W. (2022). Flavanols from nature: A phytochemistry and biological activity review. Molecules 27 (3), 719. 10.3390/molecules27030719 35163984 PMC8838462

[B11] Matatall K. A. Kadmon C. S. King K. Y. (2018). Detecting hematopoietic stem cell proliferation using BrdU incorporation. Methods Mol. Biol. 1686, 91–103. 10.1007/978-1-4939-7371-2_7 29030815 PMC6020038

[B12] McCulloch M. Ly H. Broffman M. See C. Clemons J. Chang R. (2016). Chinese herbal medicine and fluorouracil-based chemotherapy for colorectal cancer. Integr. Cancer Ther. 15 (3), 285–307. 10.1177/1534735416638738 27151587 PMC5739191

[B13] Oner A. Kobold S. (2022). Transwell migration assay to interrogate human CAR-T cell chemotaxis. Star. Protoc. 3 (4), 101708. 10.1016/j.xpro.2022.101708 36136753 PMC9508471

[B15] Quan L. Q. Hegazy A. M. Zhang Z. J. Zhao X. D. Li H. M. Li R. T. (2020). Iridoids and bis-iridoids from *Valeriana jatamansi* and their cytotoxicity against human glioma stem cells. Phytochemistry 175, 112372. 10.1016/j.phytochem.2020.112372 32305683

[B16] Rahman M. M. Rahaman M. S. Islam M. R. Rahman F. Mithi F. M. Alqahtani T. (2021). Role of phenolic compounds in human disease: Current knowledge and future prospects. Molecules 27 (1), 233. 10.3390/molecules27010233 35011465 PMC8746501

[B17] Sæbø I. P. Bjørås M. Franzyk H. Helgesen E. Booth J. A. (2023). Optimization of the hemolysis assay for the assessment of cytotoxicity. Int. J. Mol. Sci. 24, 2914. 10.3390/ijms24032914 36769243 PMC9917735

[B18] Serebriiskii I. G. Pavlov V. Tricarico R. Andrianov G. Nicolas E. Parker M. I. (2022). Comprehensive characterization of PTEN mutational profile in a series of 34,129 colorectal cancers. Nat. Commun. 13 (1), 1618. 10.1038/s41467-022-29227-2 35338148 PMC8956741

[B19] Uboveja A. Satija Y. K. Siraj F. Saluja D. (2022). p73-regulated FER1L4 lncRNA sponges the oncogenic potential of miR-1273g-3p and aids in the suppression of colorectal cancer metastasis. iScience 25 (2), 103811. 10.1016/j.isci.2022.103811 35198876 PMC8844823

[B20] Wang C. Gong X. Bo A. Zhang L. Zhang M. Zang E. (2020). Iridoids: Research advances in their phytochemistry, biological activities, and pharmacokinetics. Molecules 25 (2), 287. 10.3390/molecules25020287 31936853 PMC7024201

[B21] Xia L. Zhang B. Yan Q. Ruan S. (2018). Effects of saponins of *Patrinia villosa* against invasion and metastasis in colorectal cancer cell through NF-kappaB signaling pathway and EMT. Biochem. Biophys. Res. Commun. 503 (3), 2152–2159. 10.1016/j.bbrc.2018.08.005 30119890

[B22] Zhang T. Li Q. Li K. Li Y. Li J. Wang G. (2008). Antitumor effects of saponin extract from *Patrinia villosa* (Thunb) Juss on mice bearing U14 cervical cancer. Phytother. Res. 22 (5), 640–645. 10.1002/ptr.2354 18350512

[B23] Zhao L. Zhao H. J. Bao Y. R. Wang S. Li T. J. Meng X. S. (2019). The process optimization of extraction and purification for total flavonoids and comparative study on the efficacy of anti-liver tumors *in vitro* before and after purification. Lishizhen Med. Mater. Medica Res. 30 (3), 546–550.

[B24] Zhao Q. Kretschmer N. Bauer R. Efferth T. (2015). Shikonin and its derivatives inhibit the epidermal growth factor receptor signaling and synergistically kill glioblastoma cells in combination with erlotinib. Int. J. Cancer 137 (6), 1446–1456. 10.1002/ijc.29483 25688715

[B25] Zheng Y. Jin Y. Zhu H. B. Xu S. T. Xia Y. X. Huang Y. (2012). The anti-inflammatory and anti-nociceptive activities of *Patrinia villosa* and its mechanism on the proinflammatory cytokines of rats with pelvic inflammation. Afr. J. Tradit. Complement. Altern. Med. 9 (3), 295–302. 10.4314/ajtcam.v9i3.1 23983359 PMC3746661

