# Supplementary material for: Discovery of a heat-generated compound DHD derived from Patrinia villosa water extract with inhibitory effects on colon cancer cells viability and migration
Source: Front Chem. 2023 Jun 2;11:1195883. doi: 10.3389/fchem.2023.1195883 (PMC10272523; doi:10.3389/fchem.2023.1195883)
Supplement: Supplementary file 1 [file DataSheet1.docx]

Discovery of a heat-generated compound DHD derived from *Patrinia villosa* water extract with inhibitory effects on colon cancer cells viability and migration

Huihai Yang^1,2,#^, Tao Zheng^1,2,#^, Chuen-Fai Ku^1,2^, Cheuk Kit Ngai^3^, Grace Gar-Lee Yue^1,2^, Hung Kay Lee^3^, Clara Bik-San Lau^1,2,4,^*

**Supplementary information**


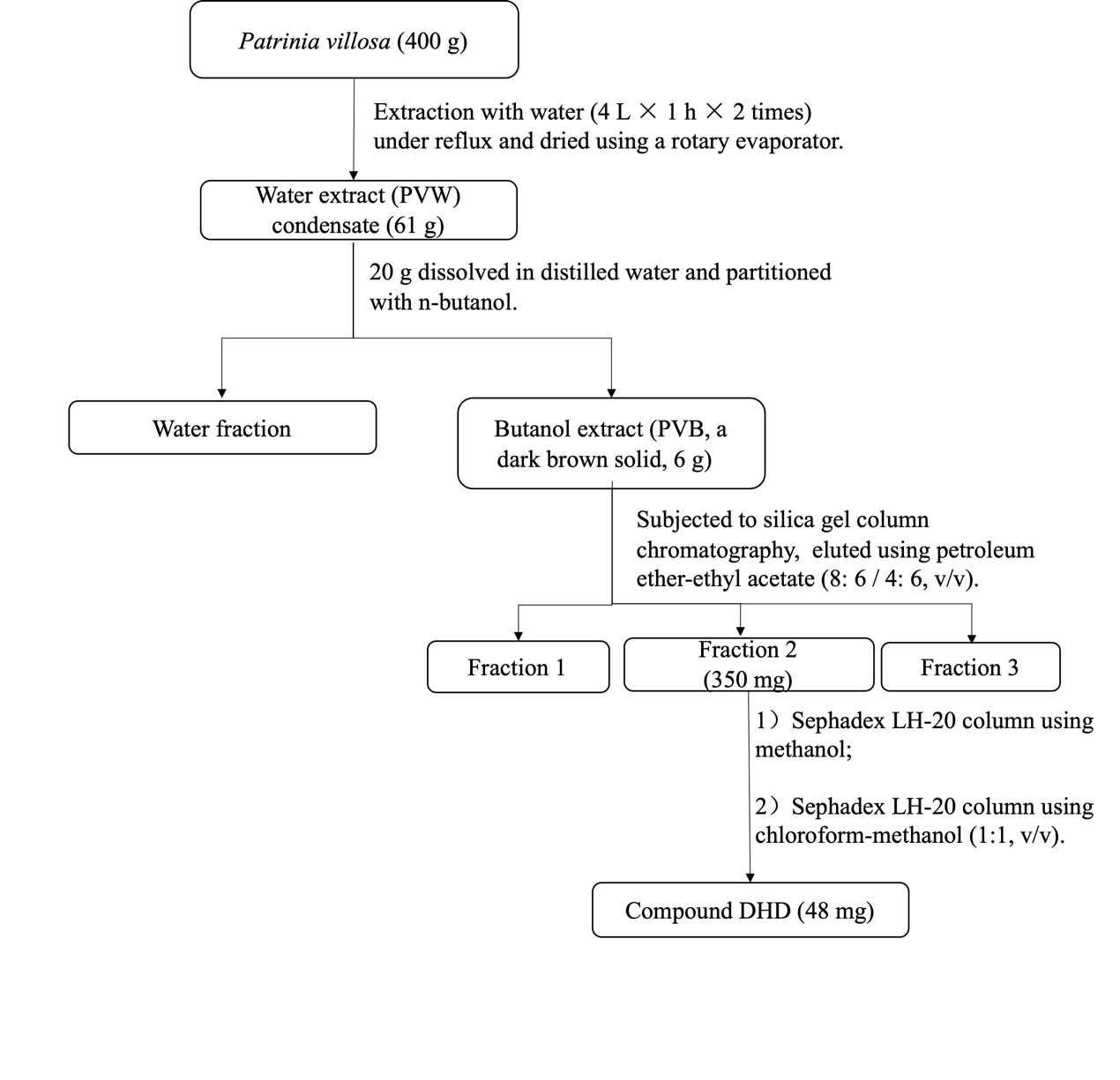


**Fig. S1.** Flow chart of extraction, fractionation and purification of DHD.

**
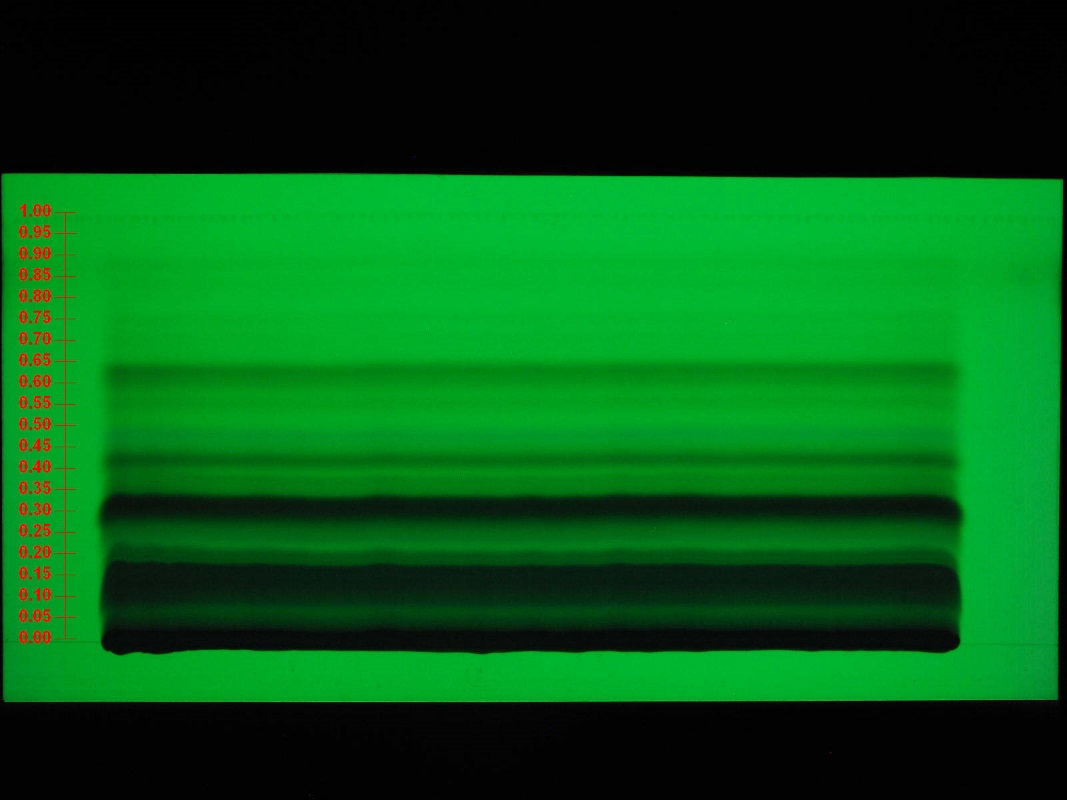
**

Fr.3

Fr.2

Fr.1

**Fig. S2.** TLC showing the three fractions (Fr.1 - Fr.3) of n-butanol part of PVW (PVB). Stationary phase: silica gel 60 F_254_; Mobile phase: chloroform: ethyl acetate = 7:3; Detection: UV254 nm

**
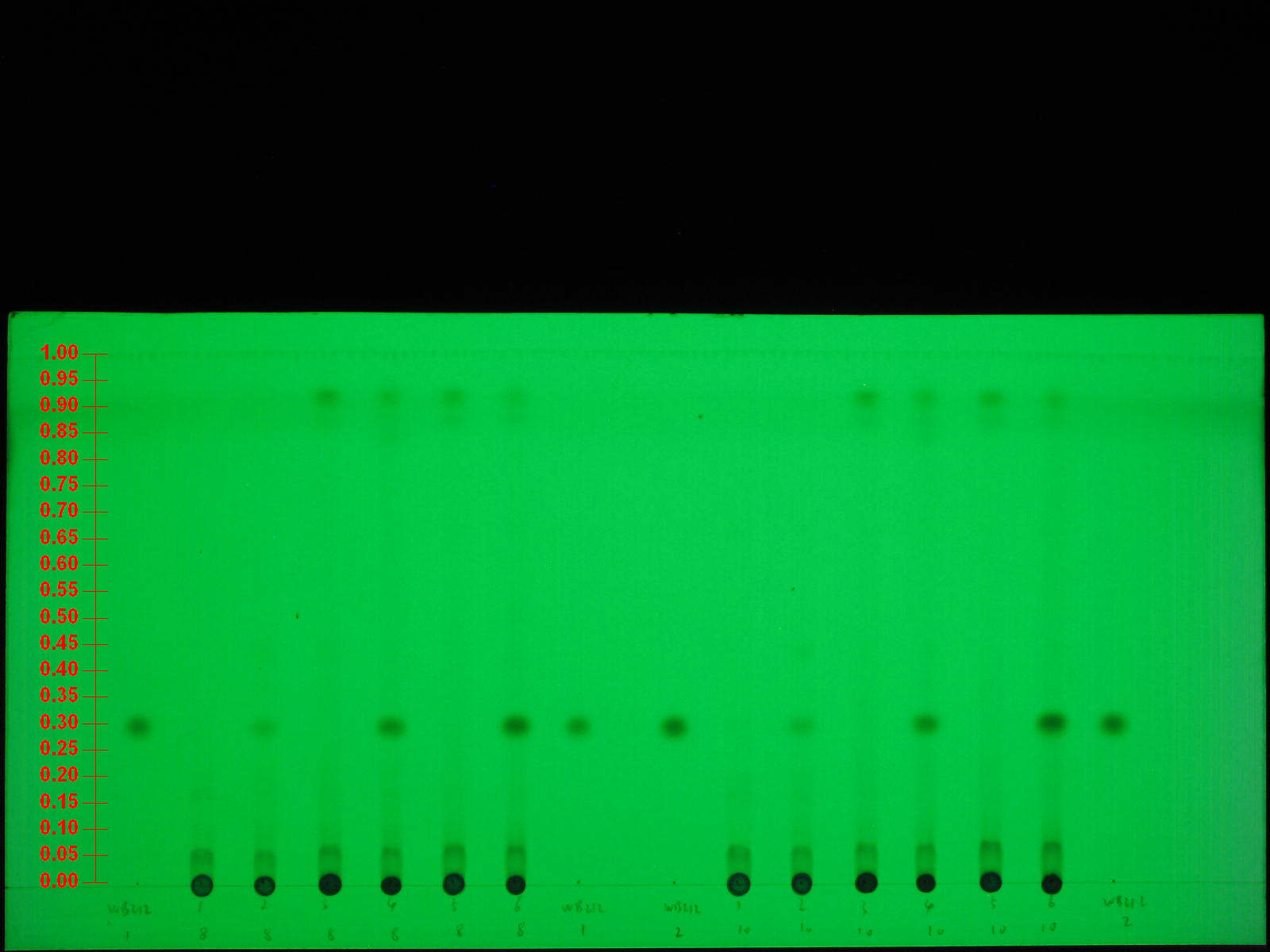
**

1 2 3 4 5

1：DHD

2：PV soaked in water at room temperature

3：PVW

4：PVE

5 : PVE + 100 % water and then reflux

**Fig. S3**: TLC analysis of different extracts from PV*.* Stationary phase: silica gel 60 F_254_; Mobile phase: chloroform: ethyl acetate = 7:3; Detection: UV254 nm


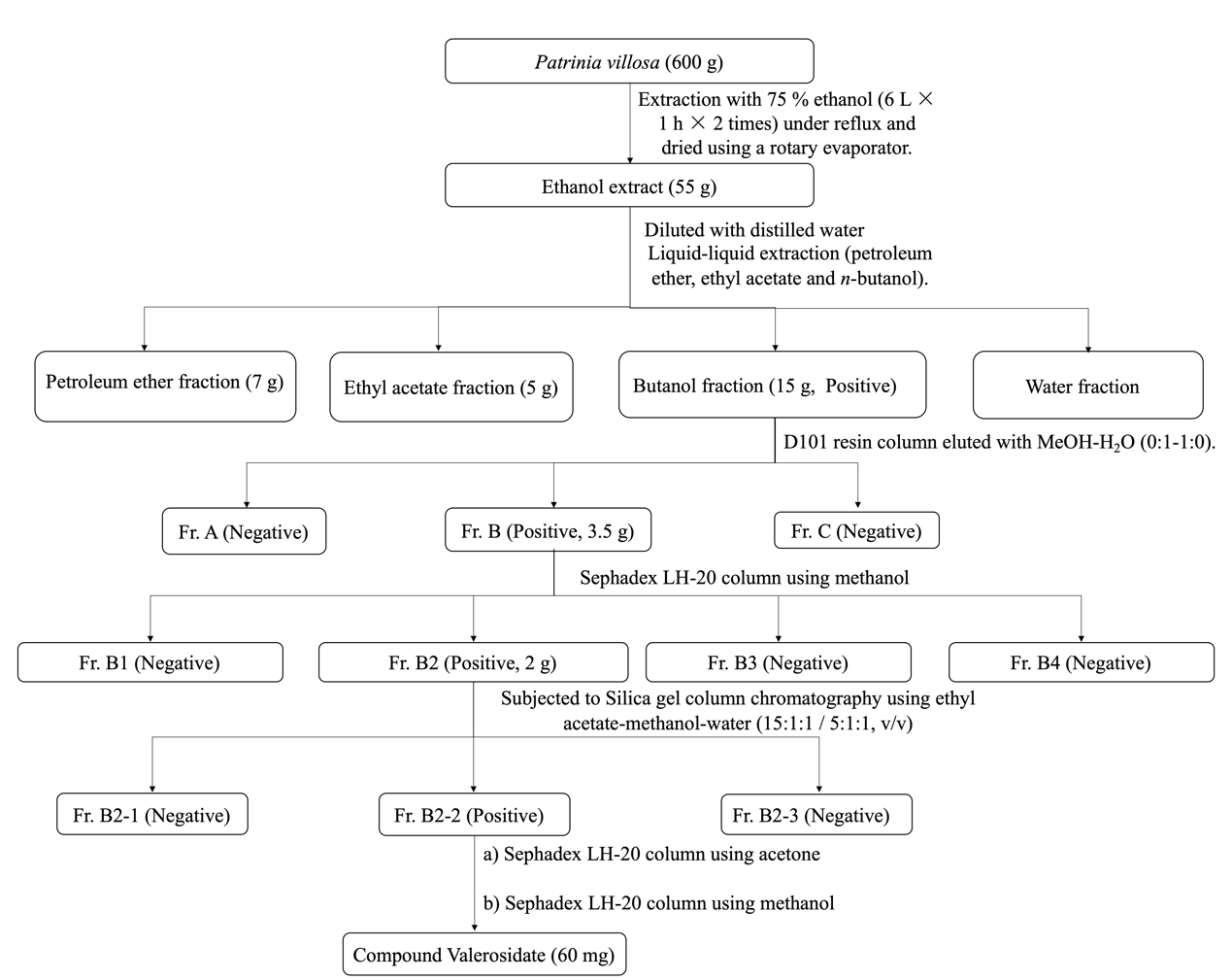


**Fig. S4.** Flow chart of extraction, tracking and purification of valerosidate.

Positive- through the tracking method, DHD was detected by TLC, i.e. the fraction contained the precursor compound.

Negative- through the tracking method, DHD was not detected by TLC, i.e. the fraction did not contain the precursor compound.

**Fig**. **S5.** ^1^H NMR spectrum of DHD (Methanol-*d*_4_, 500 MHz).

**Fig. S6.** ^13^C{^1^H} NMR spectrum of DHD (Methanol-*d*_4_, 125 MHz).

**Fig. S7.** ^1^H-^1^H COSY spectrum of DHD (Methanol-*d*_4_).

**Fig. S8.** HSQC spectrum of DHD (Methanol-*d*_4_).

**Fig. S9.** HMBC spectrum of DHD (Methanol-*d*_4_).

 **Fig. S10.** ROESY spectrum of DHD (Methanol-*d*_4_, 500 MHz).

**Fig**. **S11.** HMBC spectrum of Valerosidate (Methanol-*d*_4_).

**Fig**. **S12.** ROESY spectrum of Valerosidate (Methanol-*d*_4_, 500 MHz).

**Table S1.** Contents of DHD and valerosidate in different preparations of PVW

| PVW | Valerosidate | DHD |
| --- | --- | --- |
| Prepared by refluxing with water | <0.005% | 0.044% |
| Prepared by water maceration | 0.102% | <0.002% |

**Table S2.** Lists of antibodies used for the western blot

| **Antibodies** | **Species** | **Producers** | **Cat. No.** | **Dilution** |
| --- | --- | --- | --- | --- |
| P53 | Rabbit | Abcam | Ab32049 | 1:1000 |
| PTEN | Rabbit | Cell Signalling Technology | 9559T | 1:1000 |
| β-actin | Mouse | Sigma | A5316 | 1:3000 |
| HRP-labeled Goat  Anti-Rabbit IgG(H+L) | Goat | Cell Signalling Technology | 7074P2 | 1:3000 |
| HRP-labeled Goat  Anti-Mouse IgG(H+L) | Goat | Cell Signalling Technology | 7076P2 | 1:3000 |
